# Blocking Signaling at the Level of GLI Regulates Downstream Gene Expression and Inhibits Proliferation of Canine Osteosarcoma Cells

**DOI:** 10.1371/journal.pone.0096593

**Published:** 2014-05-08

**Authors:** Mehdi Hayat Shahi, Roseline Holt, Robert B. Rebhun

**Affiliations:** The Department of Surgical and Radiological Sciences, University of California Davis School of Veterinary Medicine, Davis, California, United States of America; Bauer Research Foundation, United States of America

## Abstract

The Hedgehog-GLI signaling pathway is active in a variety of human malignancies and is known to contribute to the growth and survival of human osteosarcoma cells. In this study, we examined the expression and regulation of GLI transcription factors in multiple canine osteosarcoma cell lines and analyzed the effects of inhibiting GLI with GANT61, a GLI-specific inhibitor. Compared with normal canine osteoblasts, real-time PCR showed that *GLI1* and *GLI2* were highly expressed in two out of three cell lines and correlated with downstream target gene expression of *PTCH1*and *PAX6*. Treatment of canine osteosarcoma cells with GANT61 resulted in decreased expression of GLI1, GLI2, PTCH1, and PAX6. Furthermore, GANT61 inhibited proliferation and colony formation in all three canine osteosarcoma cell lines. The finding that GLI signaling activity is present and active in canine osteosarcoma cells suggests that spontaneously arising osteosarcoma in dogs might serve as a good model for future preclinical testing of GLI inhibitors.

## Introduction

The Hedgehog (Hh) signaling pathway has long been known to play a critical role in early embryonic development [1]. It is now known that Hh signaling contributes to tumor growth and chemoresistance in a variety of human tumors including osteosarcoma (OSA) [2]–[7]. The Hh signaling pathway is activated upon the binding of Hh ligand to its twelve transmembrane receptor patched 1 (PTCH1). This binding relieves inhibition of smoothened (SMO), a seven transmembrane receptor regulated by PTCH1. Upon activation, SMO enters into the cytoplasm and activates transcription factors, most notably, the GLI family of proteins. In mammals, the GLI family of proteins consists of three family members, GLI1, GLI2, and GLI3 that differentially regulate downstream Hh pathways. GLI1 and GLI2 are typically found to induce distinct and overlapping target genes, whereas GLI3 can additionally serve as a repressor. GLI proteins are phosphorylated in the presence of fused serine/threonine kinase [8] and Costal-2 a kinase-like cytoplasmic protein (Cos2) [9]. Activated GLI proteins enter into the nucleus and bind to the promoter of various cell regulatory genes including GLI itself and PTCH1. Therefore, a high level of GLI expression is often indicative of activated Hh signaling.

While canonical Hh signaling is mainly mediated by PTCH1 and SMO, recent data has clearly demonstrated a parallel existence of non-canonical Hh signaling pathways [10]. The Hh signaling pathway has been found to play a critical role in many human cancers including basal cell carcinoma, medulloblastoma, glioma, colon, breast, lung, pancreas and recently in OSA [6], [7], [11]. This pathway mediates cell growth and survival through activation of pathways associated with transcription factors GLI1 and GLI2 [12], [13]. However, GLI2 often acts as a main transcription factor in the absence of GLI1 [14], [15]. Recent studies have found that human OSA cells have low expression of GLI1 compared to GLI2 and that GLI2 appears to serve as the driving transcription factor of Hh signaling thus contributing to the growth of OSA [7], [16]. A separate study also identified GLI2 as the main transcription factor of Hh signaling in human OSA growth, and further found that expression of GLI2 correlated with poor outcome in human patients with OSA [7]. Additional studies have found that inhibition of SMO in human OSA cell lines and mouse xenografts can inhibit canonical Hh signaling and may be an effective therapeutic approach for the treatment of human OSA [6].

As an alternative to targeting SMO, compounds such as arsenic trioxide have been found to inhibit GLI transcription factors and can inhibit growth of sarcoma or OSA cells that express GLI [17], [18]. Recently, two small molecules have been identified, GANT61 and GANT58, which specifically inhibit GLI proteins and serve to prevent their interaction with DNA. Subsequently it has been shown that GLI inhibitors can effectively block Hh signaling and inhibit cancer growth *in vitro* and *in vivo* [7], [19]. Targeting the Hh signaling pathway at the level of GLI, instead of at the level of SMO, may represent an attractive approach since it could potentially inhibit both canonical and non-canonical mediated up-regulation of this pathway [20].

Spontaneously occurring OSA in the dog very closely models the human disease with striking similarities at the clinical and molecular levels [21]–[23]. While very little is known about the contribution of Hh-GLI signaling in spontaneously arising canine cancers, alterations in this pathway have been implicated in both canine appendicular and extra-skeletal mammary OSA [24], [25]. Based on the importance of Hh-GLI signaling pathways in human cancers including OSA, we set out to determine whether GLI signaling is present and active in canine OSA cell lines. We found that GLI signaling is indeed present and active in multiple canine OSA cell lines and that GANT61 inhibition at the level of GLI modulates expression of downstream target genes and reduces proliferation of canine OSA cells.

## Results

### Expression of GLI1 and GLI2 in Canine OSA

**Figure 1:**
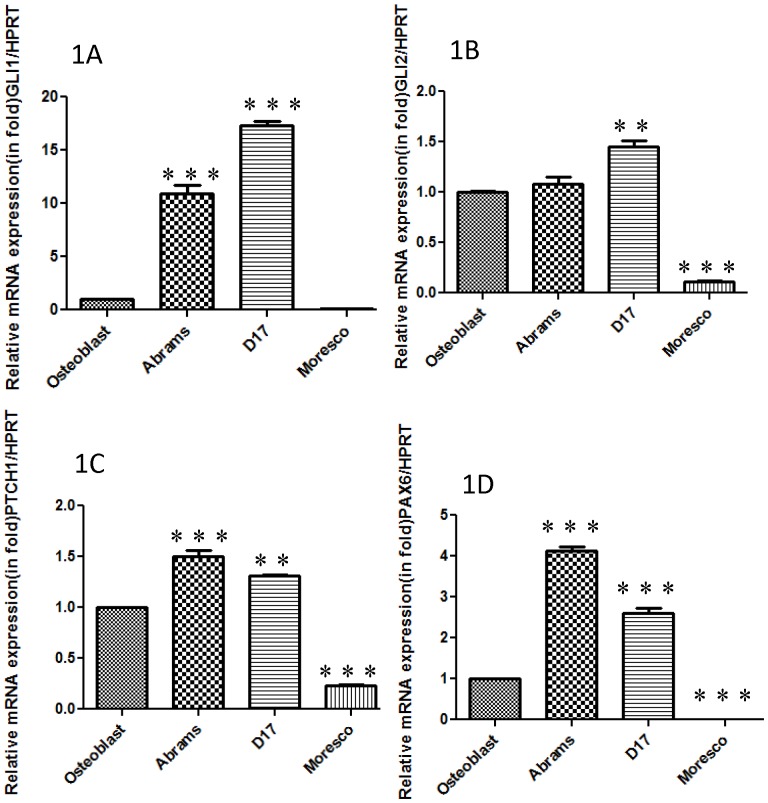
Constitutive GLI activity in canine osteosarcoma cell lines. Relative transcript expression of canine osteosarcoma cell lines and canine osteoblast cells were determined by Real Time RT-PCR. (A) Abrams and D17 cell lines showed high relative expression of *GLI1* compared to canine osteoblasts, whereas expression in the Moresco cell line was significantly decreased. (B) The D17 cell line showed high expression of *GLI2* mRNA, whereas Moresco cells expressed a significantly lower level of transcript compared to canine osteoblast cells. (C) Abrams and D17 cell lines showed high expression of *PTCH1* mRNA compared to canine osteoblast cells whereas Moresco cells had significantly lower expression. (D) Both Abrams and D17 cell lines showed high expression of *PAX6* mRNA compare to canine osteoblast cells. All mRNA expressions studies were equilibrated with the HPRT housekeeping gene. Errors bars represent S.D., statistical analysis was performed using one-way ANOVA with post-hoc Tukey's test. Significance denoted as: * p<0.05, * * p<0.01, * * * p<0.001.

**Figure 2:**
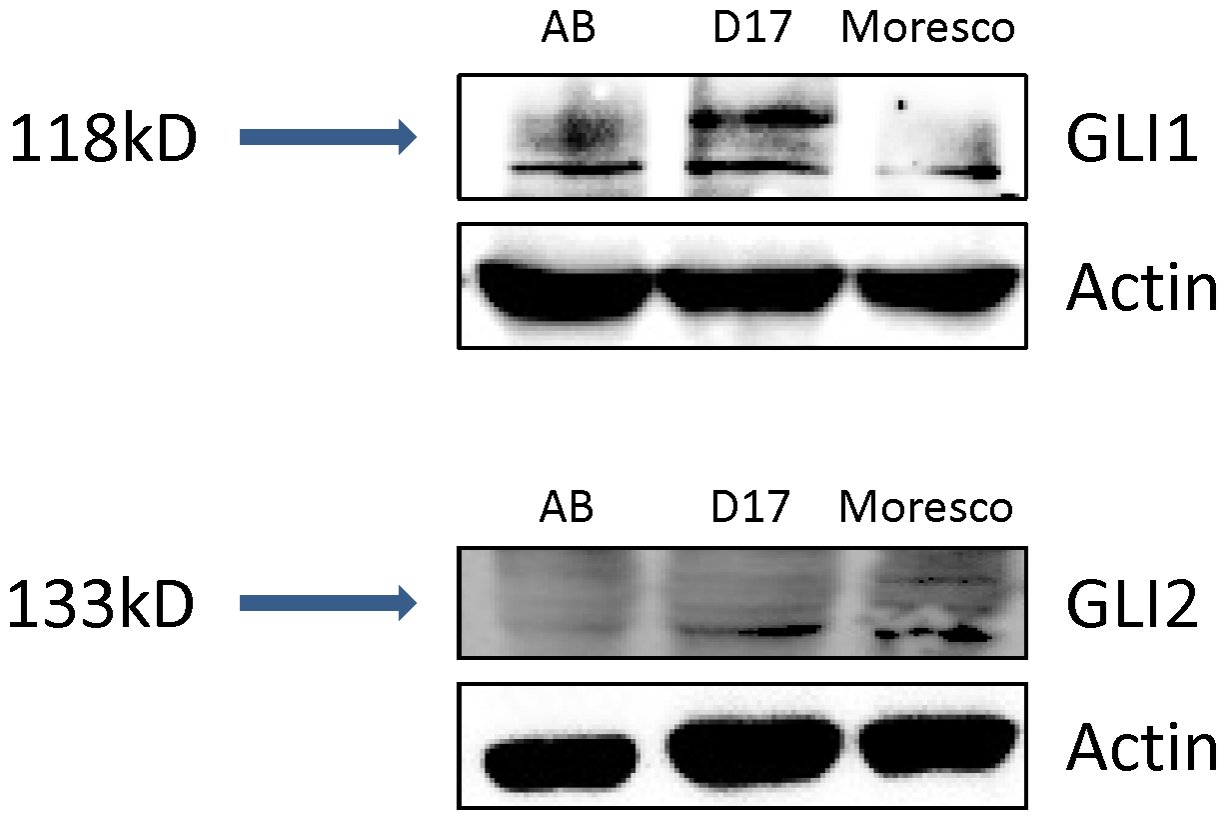
Constitutive expression of GLI proteins in canine OSA cell lines. Western Blot analyses demonstrating variable protein expression of GLI1 and GLI2 in canine OSA cell lines. Actin served as the internal loading control.

We first examined the mRNA expression of the transcription factor *GLI1* and found high expression of *GLI1* mRNA (transcript) in 2 out of 3 cell lines (Abrams and D17) relative to osteoblast cells (Fig.1A). We then checked the expression of *GLI2* at the mRNA level and also found high expression of *GLI2* mRNA in both Abrams and D17 cell lines (Fig.1B). In contrast, Moresco cells expressed relatively low levels of *GLI1* and *GLI2* mRNA when compared to osteoblasts or Abrams and D17 OSA cell lines. Western blot results confirmed varying expression of GLI1 and GLI2 proteins in all canine OSA cell lines. (Fig. 2)

### Constitutive Expression of GLI target genes in Canine OSA

We next investigated the mRNA expression of the *PTCH1* transmembrane receptor and found high expression of *PTCH1* mRNA in Abrams and D17 cell lines compared to canine osteoblast cells (Fig.1C). As would be expected based on *GLI* expression, Moresco cells expressed lower constitutive levels of *PTCH1* than osteoblast, D17, or Abrams cell lines. While little is known about PAX6 in OSA, it is a homeodomain-transcription factor I and one of the putative downstream targets of the Hh-GLI signaling pathway [26]. We therefore examined *PAX6* gene expression and found that *PAX6* was highly expressed in Abrams and D17 OSA cell lines that expressed high constitutive levels of *GLI* (Fig.1D).

### The GLI inhibitor GANT61 decreases colony formation and proliferation of canine OSA

**Figure 3:**
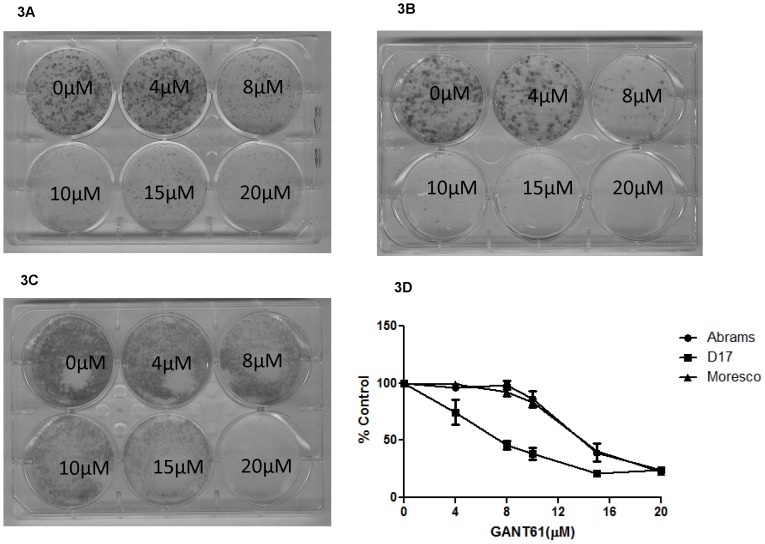
Inhibitory effects of the GLI-inhibitor GANT61 on the colony formation and viability of canine osteosarcoma cell lines. Canine OSA cell lines were treated with five different concentrations of the GLI-inhibitor GANT61. GANT61 decreased colony formation in (A) Abrams, (B) D17, and (C) Moresco cell lines. (D) GANT61 also reduced cell viability, as determined by MTS assay, in all cell lines. D17 cells appeared most sensitive to the effects of GANT61. Errors bars represent S.D.

To determine the effect of GLI inhibition on colony formation, we performed clonogenic assays in all three OSA cell lines and found that colony formation in all cell lines was inhibited by GANT61 and that D17 cells appeared to be most sensitive (Fig.3A–3C). To expand on these findings, we performed MTS assays to better define the time and concentration dependent effects of GANT61 on canine OSA cell lines (Fig. 3D). Indeed, while GANT61 inhibited proliferation of all cell lines tested, D17 cells were most sensitive to the anti-proliferative effects of GANT61.

### Expression of GLI1 and GLI2 after GANT61 treatments in D17 canine OSA cells

**Figure 4:**
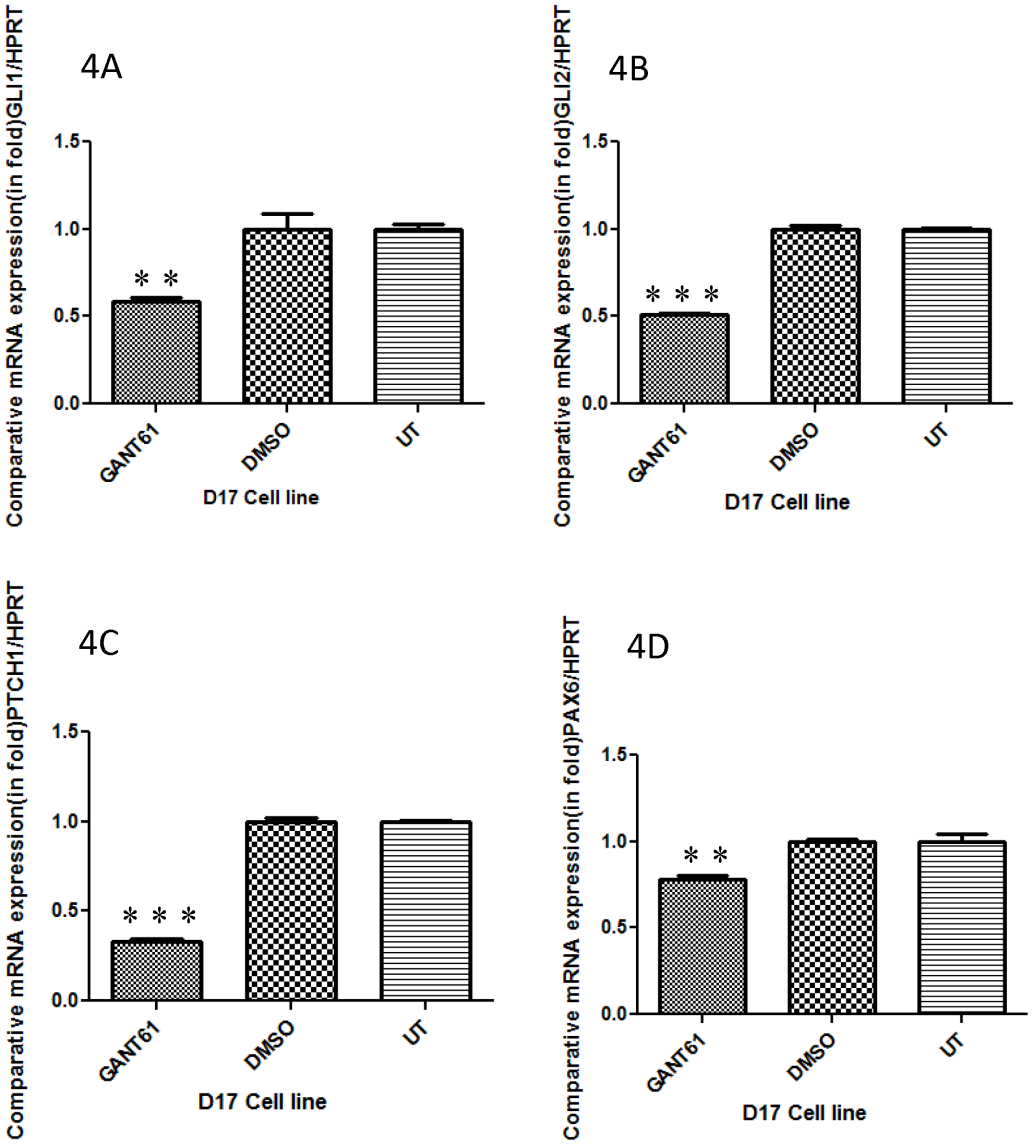
Expression of GLI1 target genes after GANT61 treatments in canine D17 cells. Canine D17 OSA cells were treated with 12 µM of GANT61 for 96 hrs and relative expression was determined by Real Time RT-PCR. (A–C) GANT61 significantly decreased expression of *GLI1, GLI2*, and *PTCH1* mRNA compared to untreated (UT) and vehicle DMSO treated cells. (D) Treatment of D17 cells with GANT61 significantly decreased expression of *PAX6* mRNA. All mRNA expression studies were equilibrated with the housekeeping gene HPRT. Errors bars represent S.D., statistical analysis was performed using one-way ANOVA with post-hoc Tukey's test and significant values denoted as: * p<0.05, * * p<0.01, * * * p<0.001.

**Figure 5:**
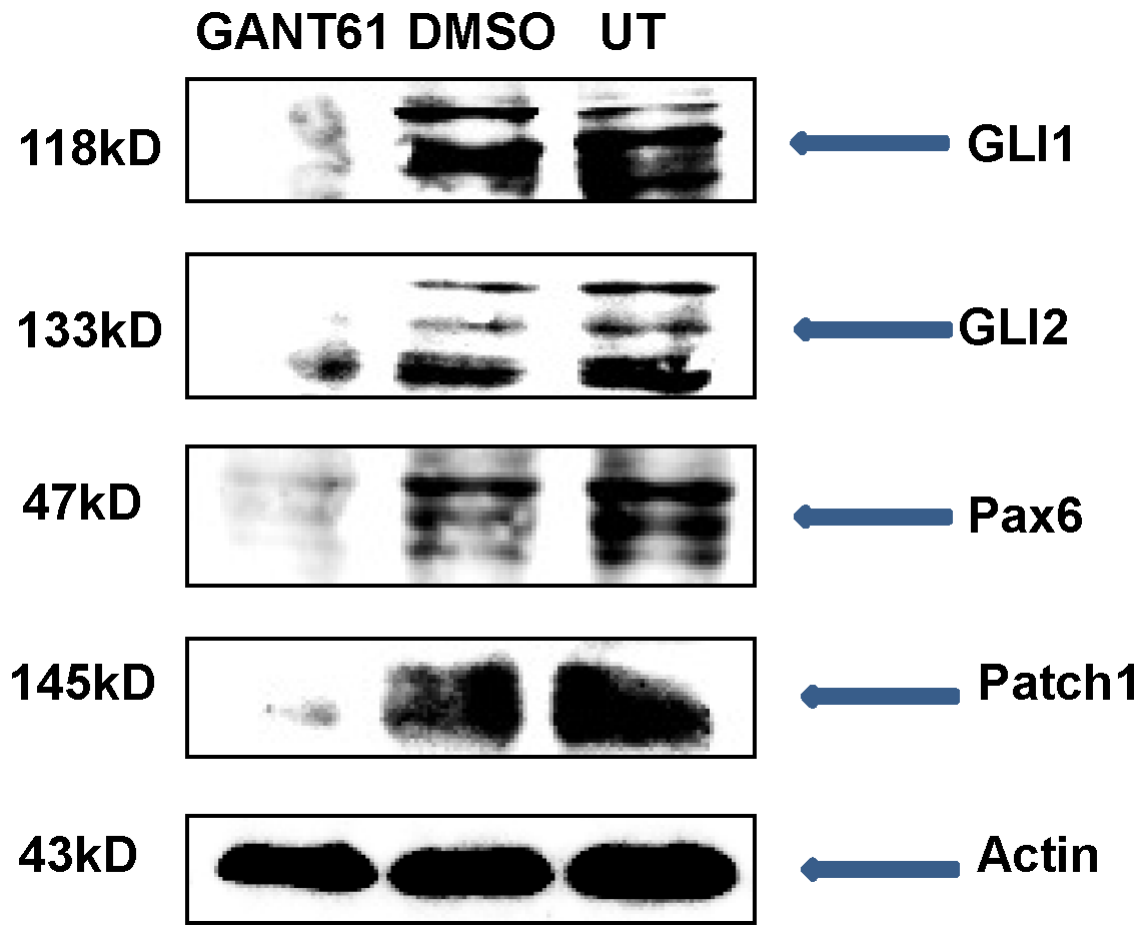
Protein expression of Hh target genes after GANT61 treatments in canine D17 cells. Canine D17 OSA cells were treated with 20 µM of GANT61 for 96 hrs and expression was determined by Western Blot analysis. GANT61 significantly decreased expression of GLI1, GLI2, PTCH1, and PAX6 compared to vehicle DMSO treated cells. Actin served as the internal loading control.

In order to further investigate the downstream effects of GLI inhibition, we initially chose to perform experiments using the D17 cell line because it expressed the highest amount of GLI proteins among the three canine OSA cell lines. After 96 hrs of GANT61 treatment (12 µM), D17 cells showed a significant reduction of *GLI1* mRNA expression compared to vehicle DMSO treated and untreated cells (Fig.4A). We also observed a significant decrease in *GLI2* mRNA expression after the same treatment with GANT61 when compared with vehicle DMSO treated and untreated cells (Fig.4B). Importantly, changes in *GLI* mRNA expression were found to correlate with changes at the protein level as determined by Western blot analyses (Fig. 5).

### Expression of GLI downstream target genes after GANT61 treatment in canine OSA cell

We next wanted to examine whether GANT61 inhibition of GLI expression led to alterations in downstream target gene mRNA and protein expression. We found that GANT61 decreased levels of *PTCH1* mRNA expression by approximately 60% in D17 cells compared to vehicle DMSO treated and untreated cells (Fig.4C). Furthermore, expression of one of the putative downstream target genes of the GLI signaling pathway, *PAX6*, showed approximately a 40% decrease in mRNA expression in canine D17 cells after GANT61 treatment compared to vehicle DMSO treated and untreated cells (Fig.4D). mRNA changes correlated with protein expression changes for GLI1, GLI2, PAX6, and PTCH1 (Fig. 5). Taken together, these data demonstrate that GLI signaling is active and indeed regulates its target genes in canine OSA cells.

### Expression of GLI1 and GLI2 in Moresco cells after GANT61 treatment

**Figure 6:**
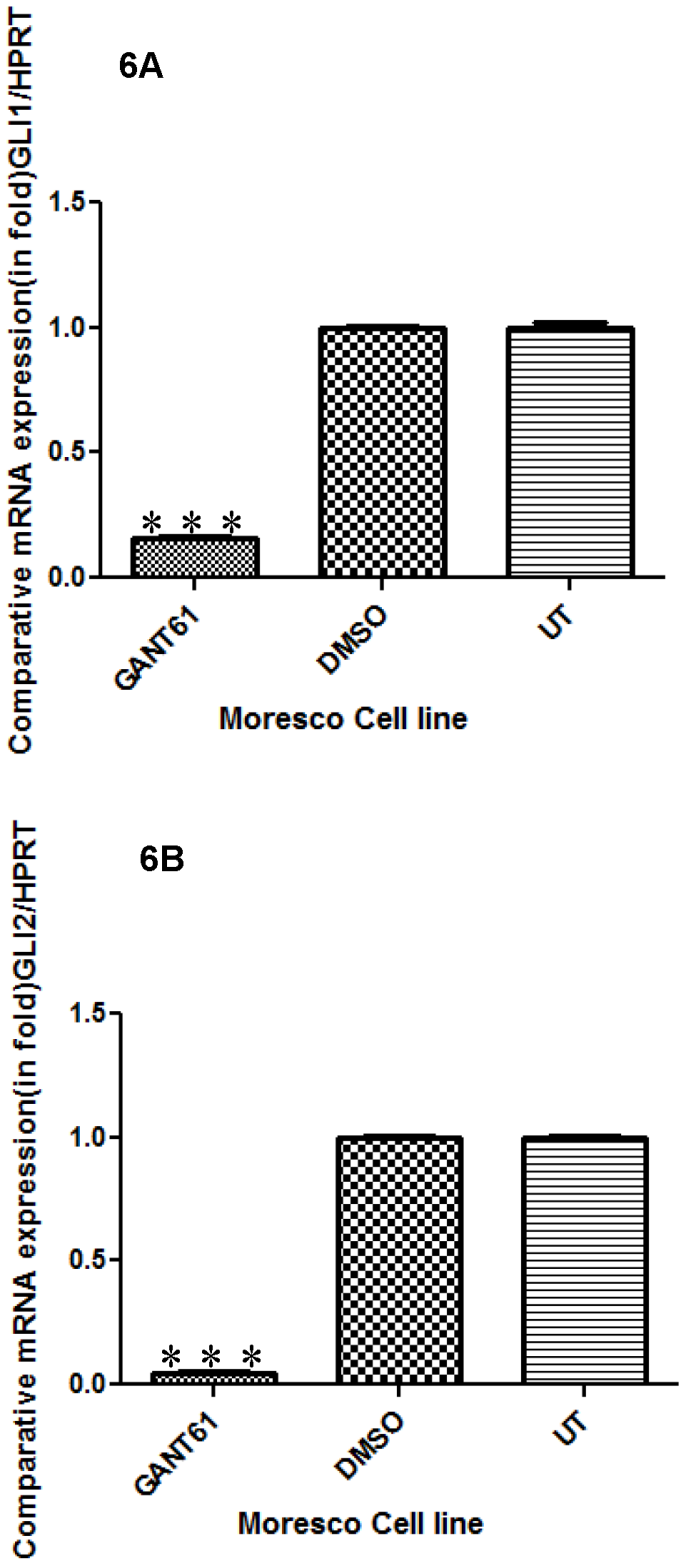
Comparative transcript expression of *GLI1 and GLI2* in canine Moresco OSA cells after GANT61 treatment. (A–B) Canine Moresco OSA cells were treated with 12 µM of GANT61 for 96 hrs and expression levels of *GLI1* and *GLI2* were determined by Real Time RT-PCR. GANT61 significantly inhibited expression of *GLI1* and *GLI2* mRNA compared to untreated (UT) or vehicle DMSO treated cells. Errors bars represent S.D., statistical analysis was performed using one-way ANOVA and post-hoc Tukey's test and significance denoted as: * p<0.05, * * p<0.01, * * * p<0.001.

Because we observed inhibition of proliferation and colony formation in Moresco cells despite relatively low constitutive expression of *GLI1* and *GLI2*, we wanted to confirm the effect of GANT61 on the mRNA expression of *GLI1 and GLI2* in Moresco cells. Despite the lower relative expression of *GLI* in Moresco cells, after 96 hrs of exposure to 12 µM of GANT61, we indeed observed a significant reduction of *GLI1 and GLI2* mRNA expression in Moresco cells (Fig. 6), confirming that even relatively low constitutive levels of GLI signaling can be inhibited.

## Discussion

Dogs with spontaneously arising OSA have been found to be an excellent model for the study of human OSA and this disease shares many clinical, genetic, and molecular similarities [21]–[23]. Recent studies have uncovered an important role for Hh-GLI signaling in human OSA and inhibition of Hh-GLI signaling has shown promising results in pre-clinical studies [6], [7], [16], [18], [27], [28]. However, no such studies have examined whether or not this pathway is active in canine OSA. We now report that multiple canine OSA cell lines express varying levels of GLI transcription factors and that the GLI inhibitor, GANT61, is capable of regulating GLI signaling and cell proliferation in canine OSA cells.

GLI transcription factors represent the primary mediators of Hh signaling. Aberrant Hh-GLI signaling has now been implicated in several human cancers, and targeting Hh has demonstrated success most notably in advanced basal cell tumors and medulloblastoma [29], [30]. The majority of studies to date that have aimed to inhibit Hh signaling have focused on inhibition of SMO with cyclopamine or small molecule inhibitors [31]. While this approach has demonstrated success in tumors with aberrant canonical PTCH1 or SMO signaling, it has now become evident that non-canonical Hh-GLI signaling may contribute to the biology of some tumors and may be independent of SMO signaling [32]. Therefore, targeting GLI could present an attractive approach for the treatment of OSA since this approach would be expected to inhibit contributions of both canonical and non-canonical Hh signaling.

While several studies have demonstrated an important role for Hh-GLI signaling in human OSA, there is little known about the role of this pathway in canine OSA. However, at least two studies implicate aberrant Hh signaling may be present in canine OSA [24], [25]. Interestingly, expression profiling in canine OSA patient samples indicated that down-regulation of Hh signaling pathways associated with poor outcome [24]. Specifically, those investigators found that significant down-regulation of gene expression for *SMO*, *PTCH2*, and *desert hedgehog* appeared to be associated with a shortened disease free interval in dogs with OSA. *GLI2* expression was not specifically identified as being aberrant in that study, however, GLI2 expression has been correlated with poor outcome in human OSA patients and appears to be the primary mediator of Hh signaling in human OSA [7], [16]. The authors are not aware of any studies previously examining expression of GLI2 in canine OSA.

Initial experiments demonstrated high constitutive expression of *GLI1* in 2 out of 3 cell lines of canine OSA compared to canine osteoblast cells. High fold expression of *GLI1* seemed indicative that GLI signaling was active and could contribute to osteo-oncogenenesis. We also observed high fold expression of *GLI2* in the same two cell lines (Abrams and D17). However, *GLI1* showed 10–15 times higher fold mRNA expression compared to *GLI2* mRNA in canine Abrams and D17 OSA cell lines. This suggested that GLI1 might possibly be the major Hh-GLI signaling regulatory transcription factor in Canine OSA. While previous studies have identified GLI1 as the main transcription factor of Hh-GLI signaling pathway in several tumor systems, two independent studies have concluded that GLI2 appears to be of highest relative importance in human OSA [7]. GANT61 is known to inhibit both GLI1 and GLI2 transcription factors [19], hence, additional studies would be required to determine the contributions of individual GLI family members in canine OSA.

We found that GANT61 was capable of inhibiting cell proliferation in all three canine cell lines but was most effective inhibiting the growth of D17 cells that expressed highest levels of GLI. Importantly, the IC50 value of GANT61 (based on reporter activity) is in the range of 5 µM [19]. Results of clonogenic assays indicated reduction in colony formation for both Abrams and D17 between 4–8 µM, whereas Moresco cells were more resistant. This same concentration range inhibited the viability of D17 cells as measured using MTS assays, however, Abrams and Moresco cells required greater than 10 µM to significantly inhibit cell viability. This discrepancy could be indicative of a differential role for GLI in colony formation/survival vs. proliferation of canine OSA. Alternatively, GANT61 inhibition of Abrams and D17 at concentrations above 10 µM on MTS assay could possibly represent off-target effects.

To further examine the effects of GANT61 on canine OSA cells, we went on to examine expression of downstream target genes before and after treatment with GANT61. The majority of studies investigating downstream target genes of GLI have focused on genes with known binding of the GLI1 consensus sequences within the promoter region. Therefore, we chose to first examine expression of constitutive GLI1 downstream target genes PTCH1 and PAX6, in canine OSA cells. We then went on to determine the effect of GANT61 on the expression of these same target genes. High expression of PTCH1 in Abrams and D17 cell lines initially supported the notion that GLI signaling was active in canine OSA. However, the Moresco OSA cell line showed low expression of *PTCH1* compared to osteoblast cells and this correlated with low expression of *GLI1* and *GLI2* expression in these cells. Importantly, *PTCH1* expression was significantly reduced in D17 cells after treatment with GANT61, supporting the notion that *PTCH1* mRNA can be regulated by the Hh-GLI pathway in canine OSA cells. These results are in agreement with previous studies showing that GLI1 knockdown decreases *PTCH1* expression in human medulloblastoma and glioblastoma cells [26].

We also found that *PAX6*, one of the putative Hh/-GLI signaling downstream target genes, showed high expression in Abrams and D17 canine OSA cell lines as would be expected from *GLI1*, *GLI2*, and *PTCH1* expression analyses. GANT61 treatment reduced 40% of the *PAX6* expression in canine OSA cells which is in agreement with a previous study wherein GLI1 knockdown reduced the expression of *PAX6* in human medulloblastoma [26]. It is important to note that medulloblastoma samples showed high expression of PAX6 [33] and it is well known that medulloblastoma is commonly associated with activation/mutation of Hh signaling pathway factors [34], [35]. Taken together, our findings indicate that GLI signaling may upregulate PAX6 in canine OSA. Examination of *PAX6* expression in osteocytes suggests that PAX6 regulates sclerostin, an osteocyte marker that inhibits canonical Wnt signaling antagonist [36]. While PAX6 is one of the critical transcription factors that regulate several genes of cell proliferation and cell patterning and migration of neuroectodermal precursor cells [37], [38], to the best of our knowledge, the role of PAX6 has not been specifically examined in OSA. Notably, it has also been reported that the *PAX6* gene can possess both proto-oncogenic and tumor suppressor characteristics and these may vary among tumor types [26]. Our result suggests PAX6 may be acting as a proto-oncogene in canine OSA, however, this remains speculative and further investigation would be needed to determine the role of PAX6 in human or canine OSA.

Altogether, the results of our studies demonstrate that 1) GLI signaling is present in canine OSA cell lines, 2) GANT61 is capable of altering expression of GLI target genes in canine OSA cells, and 3) inhibition of GLI is capable of reducing proliferation of canine OSA cells. While these findings indicate that Hh-GLI signaling is quite similar between canine and human OSA, several questions remain unanswered. Specifically, our studies have not examined the individual contribution of GLI proteins in canine OSA, whereas, GLI2 appears to be the major driver of Hh/-GLI signaling in human OSA [7], [16]. Furthermore, while the goal of these experiments was limited to determining the presence and function of GLI signaling, we did not investigate the specific effects of inhibiting Hh SMO signaling upstream of GLI. Lastly, we can make no conclusions about the clinical relevance or importance of Hh-GLI signaling in canine OSA at this time. However, our data supports the notion that canine OSA may serve as a model to explore the effects of GLI inhibition and further indicates that OSA response to Hh/-GLI inhibitors may be dependent on GLI expression. Studies are ongoing to determine the individual contributions of GLI transcription factors and to examine the expression of these proteins in canine patient tumors.

## Materials and Methods

### GLI Inhibitor

GANT61 (Catalogue #373401) was purchased from Calbiochem and dissolved in DMSO at a stock concentration of 5 mg/ml.

### Cell Cultures

Canine osteoblast cells (Cn406-05) were purchased from Cell Application Inc. Abrams and Moresco cell lines were gifts from Dr. Douglas Thamm, Department of Clinical Sciences College of Veterinary Medicine and Biomedical Sciences Colorado State University [39], [40]. D-17 (CCL-183) cell line was purchased from American Type Culture Collection (ATCC). Canine OSA cell lines were cultured at 37°C in DMEM medium (Gibco) supplemented with 10%FBS, 1% penicillin/streptomycin (Gibco), 1% MEM non-essential amino acid (cellgro) and 1% MEM vitamins (Gibco) in 5% humidified CO_2_ chamber. All cell lines tested and confirmed free from mycoplasma. Canine osteoblast cells were grown in growth medium (Cn417D-500) purchased from Cell Application Inc. under identical conditions.

### MTS Assay (Cell Proliferation Assay)

Canine OSA cell lines Abrams, D17 and Moresco were plated in triplicate at a density of 700 cells per well in 96-well plates. After 24 hours, culture medium was replaced with fresh 2% FBS medium containing various concentrations of GANT61 at concentrations between 0–20 µM for 4 days. Cell viability was measured by incubation with the 3-(4,5-dimethylthiazol-2-yl)-5-(3-carboxymethoxyphenyl)-2-(4-sulfophenyl)-2H tetrazolium, inner salt (MTS) one –solution assay reagent (CellTiter 96*, Promega) at 37°C for 1 h before reading absorbance on a spectrophotometer (SpectraMax 190, Molecular Devices) at 490 nm. DMSO was used as a vehicle to dissolve GANT61 and for controls.

### Clonogenic Assay

Cell survival was determined by clonogenic assay based on previous publication [41]. Cells were plated onto 6-well plate (2000 cells/well) allowed to adhere overnight and treated after 24 hrs with GANT61 at concentration of 0, 4, 8, 10, 15, and 20 µM and incubated for 7 days. On day 7, the colonies were washed with PBS, fixed in 100% ethanol and stained with crystal violet solution.

### Quantitative Real Time RT-PCR analysis (qRT-PCR)

Total RNA was extracted from Canine osteoblast cell line (CO), Abrams, D17 and Moresco cell lines with Tri Regent (Sigma) and chloroform-ethanol method. Then determined the quantity and quality of RNA with the help of Biophotometer (eppendorf). Total 2 µg of total RNA was converted to cDNA by using High Capacity RNA to cDNA Kit (Applied Biosystem). Transcript expression (mRNA) level of *GLI1, GLI2, PTCH1, and PAX6* was determined by using the StepOne Plus Real-Time PCR system (Applied Biosystem). Taq-Man assays (Applied Biosystem) *GLI1* (Assay ID # Cf04230663_m1) *GLI2* (Assay ID#4331348), *PTCH1* (Assay ID #Cf02690587_m1), and *PAX6* (Assay ID#Cf02675240_m1) were used to determine the expression. All transcripts expression was equilibrated with endogenous control HPRT1 (Assay ID#Cf02626256_m1) expression (Figure S1, S2, S3). The real time expression calculation is based on Delta-delta (ΔΔC) method by PE Applied Biosystem (Perkin Elmer, Forster City, CA)[42].

### Western blot analysis

The D17 cell line was treated with 20 µM GANT61 or vehicle DMSO for 96 hrs and cell lysate was made in lysis buffer. Protein was quantified using a BCA protein Assay Reagent (bicinchonic acid) (Pierce). A total of 40 µg of protein was loaded per sample in the 7.5% polyacrylamide gels under denaturing and reducing conditions and protein was transferred to nitrocellulose membranes. After transfer of protein, the membrane was probed and incubated overnight at 4°C with antibodies Rabbit anti-GLI1 (Abcam ab49314), Rabbit anti-GLI2 (Abcam ab26056), Rabbit anti-Patch1(Sigma-Aldrich P0088), Rabbit anti-Pax6 (Abcam ab5790), and Mouse anti-Actin (Santa Cruz sc-56459) in 5% non-fat milk. Then membranes were washed and subsequently exposed to the appropriate HRP-conjugated secondary antibodies for 1 hr. at room temperature. Bands were visualized by the enhanced chemiluminescence (Pierce) in Fluorchem E Imaging system (Protein Simple, CA). Constitutive expression levels of GLI1 and GLI2 were performed similarly, in the absence of treatment for Abrams, D17 and Moresco cell lines.

### Statistical analysis

All results shown are representative of experiments done in triplicate. Analyses were performed using one-way ANOVA and post-doc Tukey's testing (GraphPad Prism version 5.0). Differences were considered significant with a p<0.05.

## Supporting Information

Figure S1 **Amplification plot of** ***GLI1*** **,** ***GLI2*** **,** ***PTCH1*** **and** ***PAX6*** **RNA expression in canine OSA cell lines: This plot was used to determine the Cycle threshold (Ct)value; PCR Cycle number as X and the mean ΔRn (an algorithm compared the amount of the TaqMan assay reporter dye emission (R) with the quenching dye emission (Q) during the Real Time PCR amplification process) value as Y.** The standard ΔRn (Y = 0.05) of exponential phase of amplification was selected to determine the optimal CT value. Less cycle number to reach exponential phase of amplification indicates high copy number of RNA (less Ct value). (A) Amplification plot of *GLI1* in Canine osteoblast (CO), Moresco, Abrams and D17. (B) Amplification plot of *GLI2* in Canine osteoblast (CO), Moresco, Abrams and D17. (C) Amplification plot of *PTCH1* in canine CO, Moresco, Abrams and D17. (D) Amplification plot of *PAX6* in canine CO, Moresco, Abrams and D17.
(TIF)

Figure S2 **Amplification plot of** ***GLI1*** **,** ***GLI2*** **,** ***PTCH1*** **and** ***PAX6*** **mRNA expression in canine cell line D17 after the treatment of GANT61 and DMSO.** (A) Amplification plot of *GLI1* expression in Canine OSA D17 cell line compared to GANT61 and DMSO (control). (B) Amplification plot of *GLI2* expression in D17 cell line compared to GANT61 and DMSO (control). (C) Amplification plot of *PTCH1* in canine OSA cell line D17 compared to GANT61 and DMSO treatment. (D) Amplification plot of *PAX6* in canine OSA cell line D17 compared to GANT61 and DMSO treatment. GANT61 treated cells showed increased number of cycle to reach exponential phase of amplification compare to DMSO (control) (decreased mRNA copy number in GANT61 treated cells compared to DMSO).
(TIF)

Figure S3 **Amplification plot of** ***GLI1*** **and** ***GLI2*** **mRNA expression in canine cell line Moresco after the treatment of GANT61 and DMSO.** (A) Amplification plot of *GLI1* expression in Canine OSA Moresco cell line after the treatment GANT61 and DMSO (control). (B) Amplification plot of *GLI2* expression in Canine OSA Moresco cell line after the treatment of GANT61 and DMSO (control). GANT61 treated cells showed increased number of cycle to reach exponential phase of amplification compare to DMSO (control) (decreased mRNA copy number in GANT61 treated cells compared to DMSO).
(TIF)
