# Supplementary figures and images for: Blocking Signaling at the Level of GLI Regulates Downstream Gene Expression and Inhibits Proliferation of Canine Osteosarcoma Cells
Source: PLoS One. 2014 May 8;9(5):e96593. doi: 10.1371/journal.pone.0096593 (PMC4014515; doi:10.1371/journal.pone.0096593)

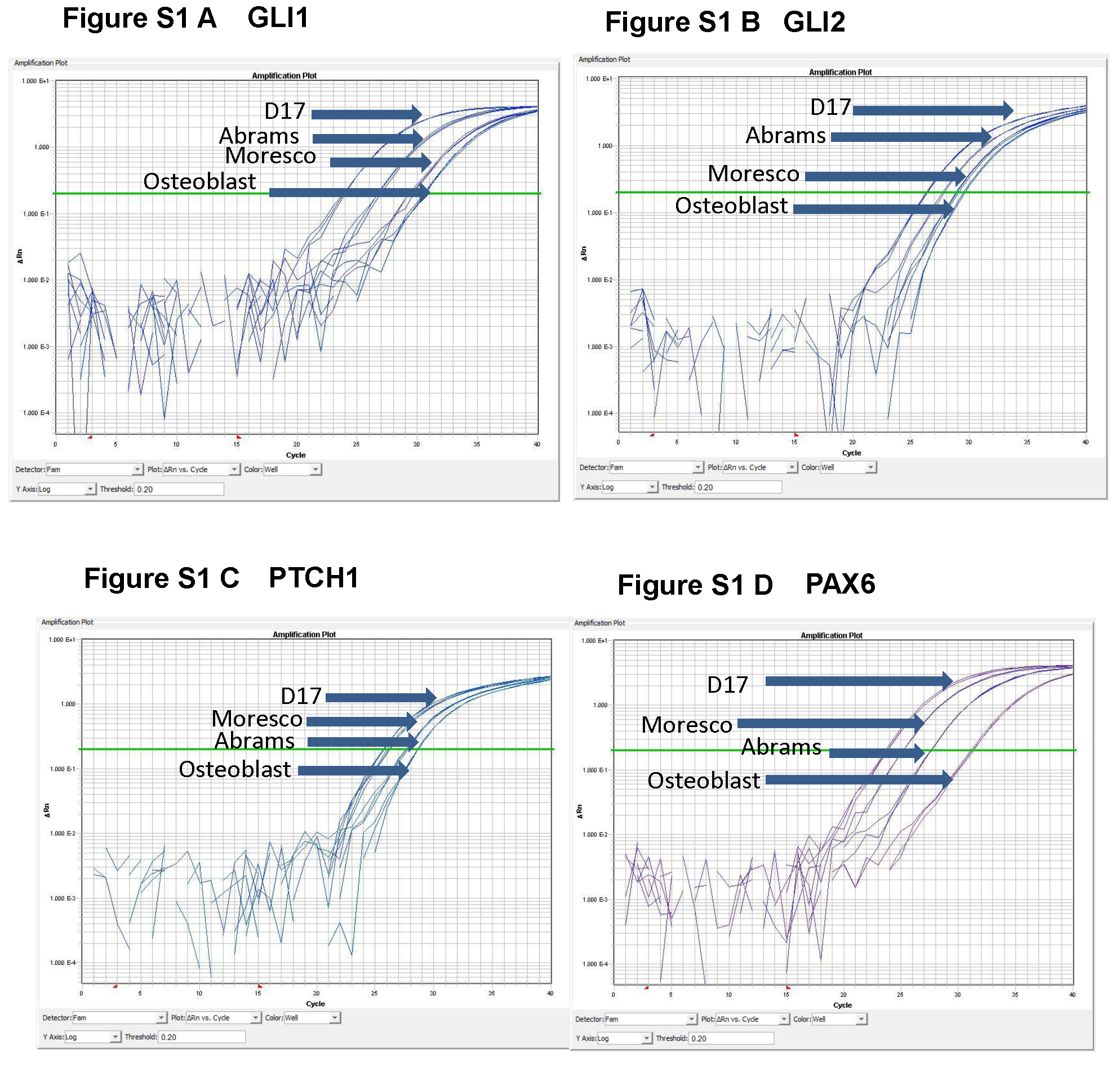

Supplement: Figure S1 — Amplification plot of GLI1 , GLI2 , PTCH1 and PAX6 RNA expression in canine OSA cell lines: This plot was used to determine the Cycle threshold (Ct)value; PCR Cycle number as X and the mean ΔRn (an algorithm compared the amount of the TaqMan assay reporter dye emission (R) with the quenching dye emission (Q) during the Real Time PCR amplification process) value as Y. The standard ΔRn (Y = 0.05) of exponential phase of amplification was selected to determine the optimal CT value. Less cycle number to reach exponential phase of amplification indicates high copy number of RNA (less Ct value). (A) Amplification plot of GLI1 in Canine osteoblast (CO), Moresco, Abrams and D17. (B) Amplification plot of GLI2 in Canine osteoblast (CO), Moresco, Abrams and D17. (C) Amplification plot of PTCH1 in canine CO, Moresco, Abrams and D17. (D) Amplification plot of PAX6 in canine CO, Moresco, Abrams and D17. (TIF) [file pone.0096593.s001.tif]

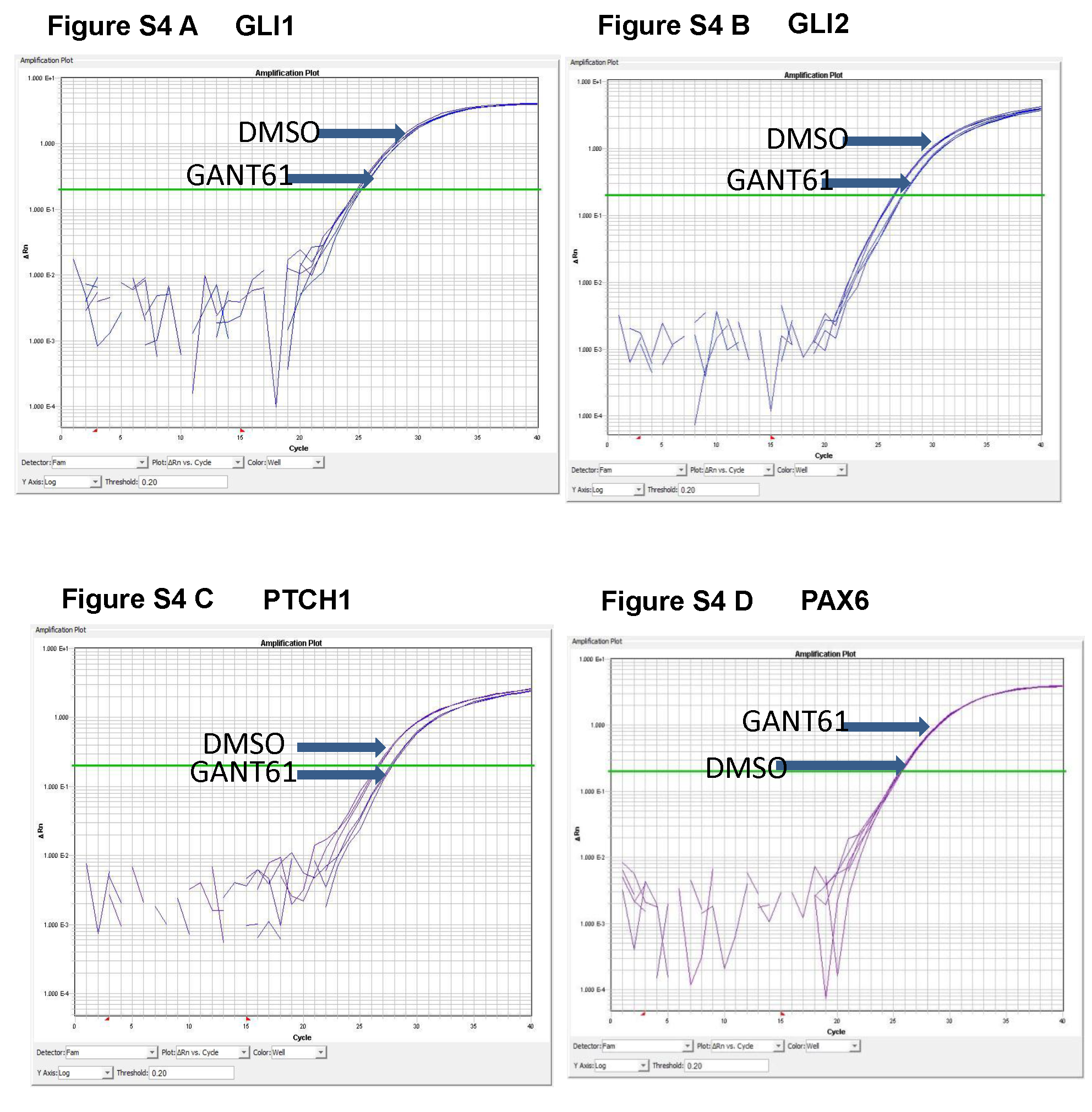

Supplement: Figure S2 — Amplification plot of GLI1 , GLI2 , PTCH1 and PAX6 mRNA expression in canine cell line D17 after the treatment of GANT61 and DMSO. (A) Amplification plot of GLI1 expression in Canine OSA D17 cell line compared to GANT61 and DMSO (control). (B) Amplification plot of GLI2 expression in D17 cell line compared to GANT61 and DMSO (control). (C) Amplification plot of PTCH1 in canine OSA cell line D17 compared to GANT61 and DMSO treatment. (D) Amplification plot of PAX6 in canine OSA cell line D17 compared to GANT61 and DMSO treatment. GANT61 treated cells showed increased number of cycle to reach exponential phase of amplification compare to DMSO (control) (decreased mRNA copy number in GANT61 treated cells compared to DMSO). (TIF) [file pone.0096593.s002.tif]

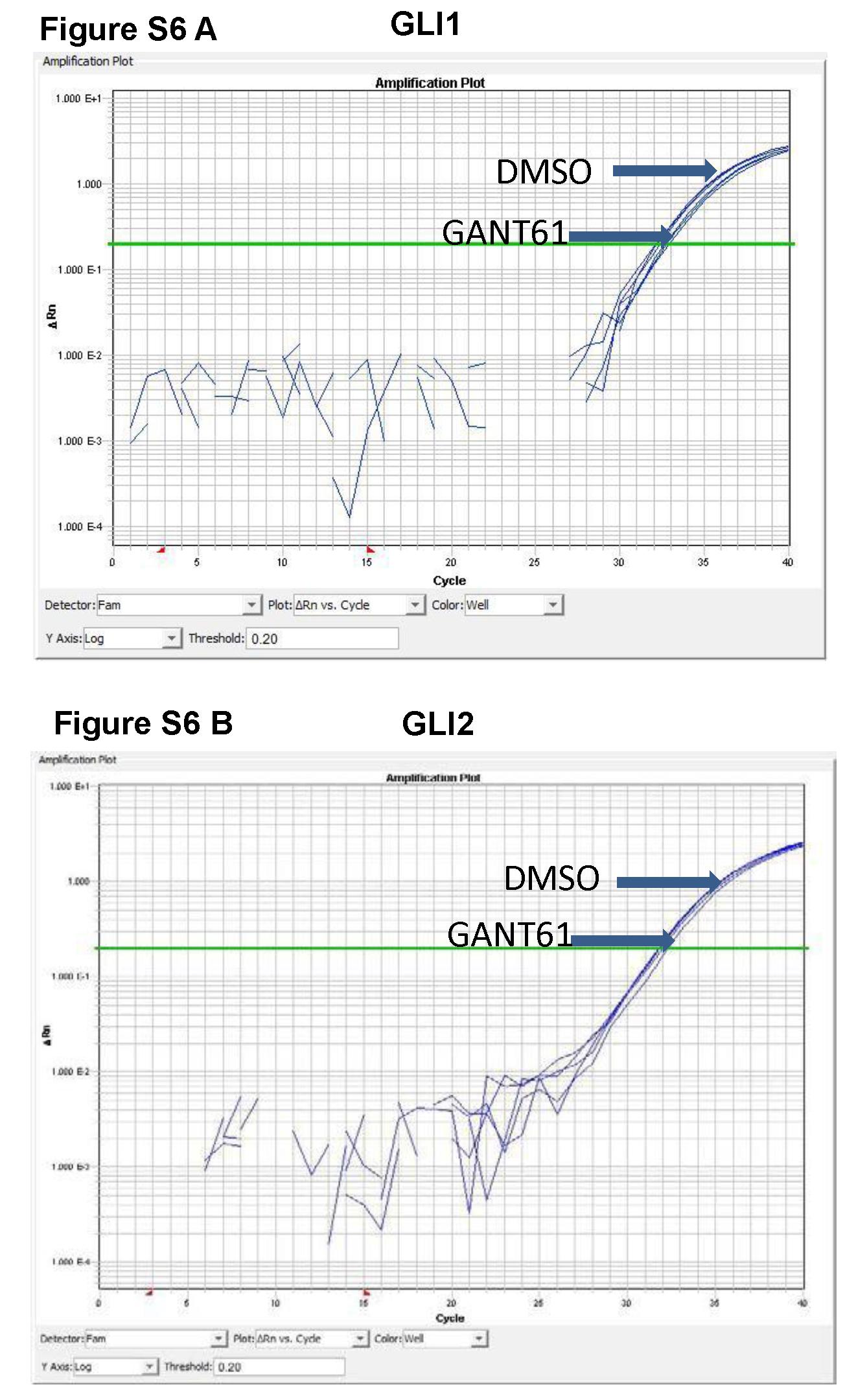

Supplement: Figure S3 — Amplification plot of GLI1 and GLI2 mRNA expression in canine cell line Moresco after the treatment of GANT61 and DMSO. (A) Amplification plot of GLI1 expression in Canine OSA Moresco cell line after the treatment GANT61 and DMSO (control). (B) Amplification plot of GLI2 expression in Canine OSA Moresco cell line after the treatment of GANT61 and DMSO (control). GANT61 treated cells showed increased number of cycle to reach exponential phase of amplification compare to DMSO (control) (decreased mRNA copy number in GANT61 treated cells compared to DMSO). (TIF) [file pone.0096593.s003.tif]
